# Characterization of a New Model of Thromboembolic Stroke in C57 black/6J mice

**DOI:** 10.1007/s12975-013-0315-9

**Published:** 2013-12-19

**Authors:** Saema Ansar, Eva Chatzikonstantinou, Anja Wistuba-Schier, Silvia Mirau-Weber, Marc Fatar, Michael G. Hennerici, Stephen Meairs

**Affiliations:** Department of Neurology, Universitätsmedizin Mannheim, Heidelberg University, Theodor-Kutzer-Ufer 1-3, 68167 Mannheim, Germany

**Keywords:** Stroke models, Thromboembolic stroke, C57 black/6J mice

## Abstract

This study characterizes a new model of thromboembolic stroke of the middle cerebral artery in C57 black/6J mice, thus offering an opportunity to use the model for studying ischemic stroke in transgenic mice. Thromboembolic stroke was induced by local injection of either 1.5 or 3.0 UI of thrombin directly into the right MCA of C57 black/6J mice. Cerebral blood flow (CBF) velocity was measured continuously by laser Doppler flowmetry, which allowed documentation of both MCA occlusion and of spontaneous recanalization. After 24 h, all animals were euthanized. Cryosections were cut at 400-μm intervals and silver stained with the high-contrast method for volumetric assessment of infarct size. Interleukin (IL)-6, tumor necrosis factor-alpha (TNF-α), caspase-3 and hsp 70 protein levels were investigated by immunofluorescence. Thrombin injection resulted in clot formation in all animals. Cortical infarction occurred in 63 % of the mice while 37 % had a spontaneous MCA recanalization during the first 20 min following thrombin injection. In cases of successful MCA occlusion with consequent infarction, the clot was stable up to 2 h after formation. Subsequently, 20 % recanalized spontaneously. Infarctions were restricted to the cortex with a mean lesion volume of 36 ± 5 for 1.5 UI and 56 ± 8 for 3.0 UI thrombin. Protein levels of IL-6, TNF-α, caspase-3, and hsp 70 were significantly increased after MCAO. The results demonstrate that the mouse thromboembolic stroke model produces cortical infarctions of consistent size in C57 black/6J mice, which is dependent upon the amount of thrombin used for clot formation. Spontaneous MCA recanalization occurs after 2 h of ischemia in 20 % of mice. Thus, the thromboembolic model is an applicable stroke model for C57 black/6J mice, which mimics many of the features of human stroke, including spontaneous recanalization. However, strain differences between Swiss and C57 black/6J mice must be taken into account when using the model.

## Introduction

Although numerous treatment strategies have shown a beneficial effect in animal models of cerebral ischemia, very few have been shown to improve outcome in human phase III trials [20]. Indeed, the outcome of statistically reliable prospective phase III trials was positive in only 3 out of 11 thrombolytic interventions and in none of 19 neuroprotection trials [13]. Even the Stroke Acute Ischemia NXY-059 (SAINT) trial failed to show any efficacy, despite convincing preclinical and phase IIb data [23]. Apart from a multitude of factors related to poor study design [17], the animal model itself may contribute to this lack of translation through its inability to adequately represent the pathophysiology of naturally occurring cerebral ischemia. Different models of experimental focal cerebral ischemia are known to exhibit different pathophysiologies, which respond differently to therapeutic interventions but which are of variable relevance to the clinical setting [11].

A number of stroke models have been used in a variety of species [3, 6, 11, 15, 16]. The intraluminal suture transient MCA occlusion (MCAO) model is the most frequently used model as it produces much lower mortality than the permanent MCAO model, provides reproducibility and allows reperfusion. Its relevance to naturally occurring stroke in humans, however, is questionable. Indeed, it was recently recommended that this model should be eliminated from the repertoire of preclinical stroke research [13], because it leads to a secondary injury developing after a free interval of as long as 6–12 h, which does not reflect the true pathophysiology of stroke [13]. The thromboembolic stroke model mimics human stroke more closely than other models, since most of the human strokes are caused by thromboembolism [6, 15]. However, these models have accomplished limited significance in the stroke research field due to their variability in producing accurate and reproducible infarct volumes [19, 28]. In addition, the mortality rate is high [1]. Thus, there remains a need to develop appropriate animal models for stroke preclinical research.

Recently, a novel mouse model based on an in situ thromboembolic occlusion of the middle cerebral artery (MCA) was described that demonstrated a precise and reproducible infarct volume with low mortality rate [21]. In this study, we characterize the adaptation of this model to C57 black/6J mice, a mouse strain offering the advantage of investigating stroke pathophysiology in transgenic mice.

## Materials and Methods

All animal procedures were carried out strictly within national laws and guidelines and were approved by the Ethical Committee for Laboratory Animal Experiments at the state council in Karlsruhe, Germany. All institutional and national guidelines for the care and use of laboratory animals were followed.

### Thromboembolic Stroke

**Fig. 1:**
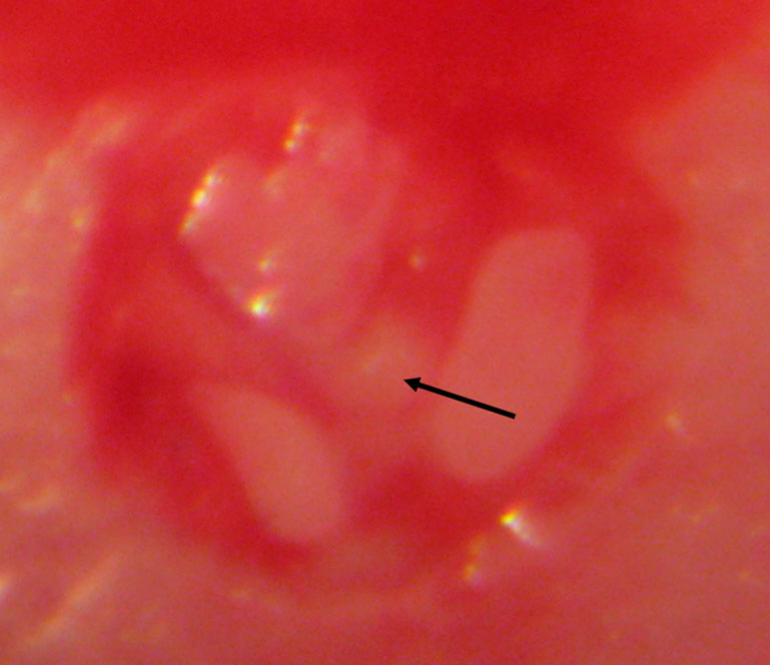
Illustration of the occlusion of the MCA. In situ thrombin injection in the bifurcation and subsequent clot formation. The branches of the MCA become *dark red* because of the obstructed blood flow and a *white clot* becomes visible in the bifurcation of the MCA

Thromboembolic stroke was induced by local injection of thrombin directly into right MCA of mice as originally described by Orset et al. [21]. Briefly, male C57 black/6J mice (20–30 g) were anesthetized with 5 % isoflurane and thereafter maintained with 1–2 % isoflurane during the surgical procedure. An electric temperature probe was inserted into the rectum of the mouse to record body temperature, which was maintained at 37 °C with a heat pad. The mice were placed in a stereotaxic device and the temporal muscle was retracted. A small craniotomy was performed, the dura was excised, and the middle cerebral artery (MCA) was exposed. The laser Doppler flow probe for measurement of cortical CBF was placed on the skull in the MCA territory with a drop of glue. Finally, a micropipette filled with 1 μl of purified murine alpha-thrombin (1.5 or 3 UI) was introduced into the lumen of the MCA bifurcation and injected carefully to induce the formation of a clot in situ (Fig. 1). The pipette was removed 10 min after the injection at which time the clot had stabilized. CBF velocity was measured continuously by laser Doppler flowmetry allowing determination of spontaneous clot dissolution. A clot was defined as successful when the cerebral blood flow velocity decreased to a minimum of 60 % from baseline at the time of thrombin injection and remained stable. CBF was measured throughout the duration of the experiment. After 24 h mice were euthanized and the brains were removed and frozen in isopentane.

### Quantification of Infarct Size

Postmortem, 10-μm coronal cryosections were cut at 400-μm intervals and stained with high contrast silver infarct method [27]. Focal edema in the ischemic hemisphere was determined according to Swanson et al. [26]. Photographs of each coronal section were taken and analyzed using ImageJ.

### Histology Examination

Hematoxylin–eosin–saffron staining was carried out for further neuropathological analysis. The neurons in the ischemic area were classified as intact or necrotic. The neurons were classified as necrotic when the cell was swelling and showed karyolysis, pyknosis, karyorhexis, cytoplasmic eosinophilia or loss of affinity for hematoxylin [7, 14, 24]. The area of severe ischemic neuronal damage (necrotic) was expressed as percentage of the total area of the brain and corrected for the presence of edema [5].

### Immunohistochemistry

For immunohistochemistry, the indirect immunofluorescence method was used. The brains were removed and frozen in ice-cold isopentane. They were then sectioned into 10-μm-thick slices in a cryostat. The cerebral cryosections were fixed for 10 min in ice-cold acetone and thereafter rehydrated in phosphate buffer solution (PBS) containing 0.25 % Triton X-100 for 15 min. The tissue was then permeabilized and blocked for 1 h in blocking solution containing PBS, 0.25 % Triton X-100, 1 % BSA and 5 % normal donkey serum. The sections were incubated over night at 4 °C with the following primary antibodies: rabbit polyclonal IL-6 (Abcam, ab6672), diluted 1:200, rabbit polyclonal hsp 70 (Abcam, ab2787), diluted 1:50, rabbit polyclonal caspase 3 (Abcam, ab4051), diluted 1:50, rabbit polyclonal TNF-α (Abcam, ab6671), diluted 1:200. All dilutions were done in PBS containing 0.2 % Triton X-100, BSA 1 and 2 % normal goat serum. Sections were subsequently washed with PBS and incubated with secondary antibody for 1 h at room temperature. The secondary antibody used was goat-anti-rabbit Alexa 488 conjugated (Invitrogen), diluted 1:400 in PBS containing 0.2 % TritonX-100 and BSA 1 %. In addition, double staining with DAPI (KPL) 1:5,000 was performed. The sections were washed subsequently with PBS and mounted with mounting medium (Moviol). The same procedure was used for the negative controls but primary antibodies were omitted. The immunoreactivity of the antibodies were visualized and photographed with a Nikon confocal microscope A1R fitted with fluorescence optics at the appropriate wavelength.

Images were analyzed using the ImageJ software (http://rsb.info.nih.gov/ij/). The fluorescence in different areas in each section was measured and a mean value was calculated. The same ROI was used for the ipsilateral and contralateral sides. These values are presented as percentage fluorescence in the ipsilateral compared to the contralateral group, where the contralateral side is set to 100 %.

### Statistics

Data are expressed as mean ± standard error of the mean (s.e.m.), and *n* refers to the number of mice. Statistical analyses were performed with Kruskal–Wallis non-parametric test with Dunn's post-hoc test, where *P* < 0.05 was considered significant.

## Results

### Thromboembolic Stroke Model

**Fig. 2:**
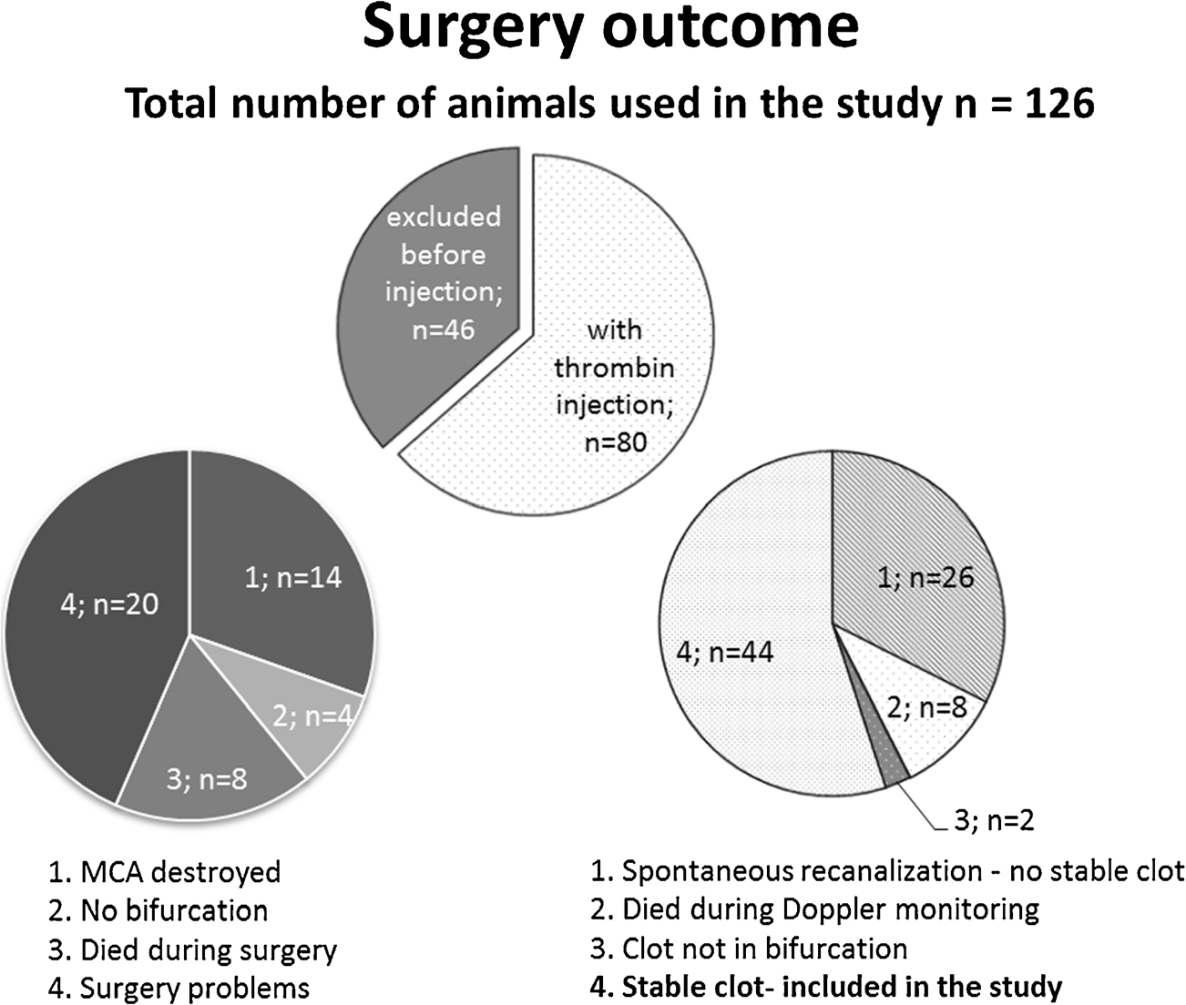
Detailed description of the experiments and surgery outcome

**Fig. 3:**
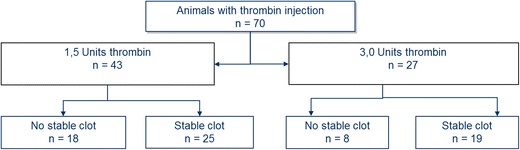
Flow chart of the experiments

**Fig. 4:**
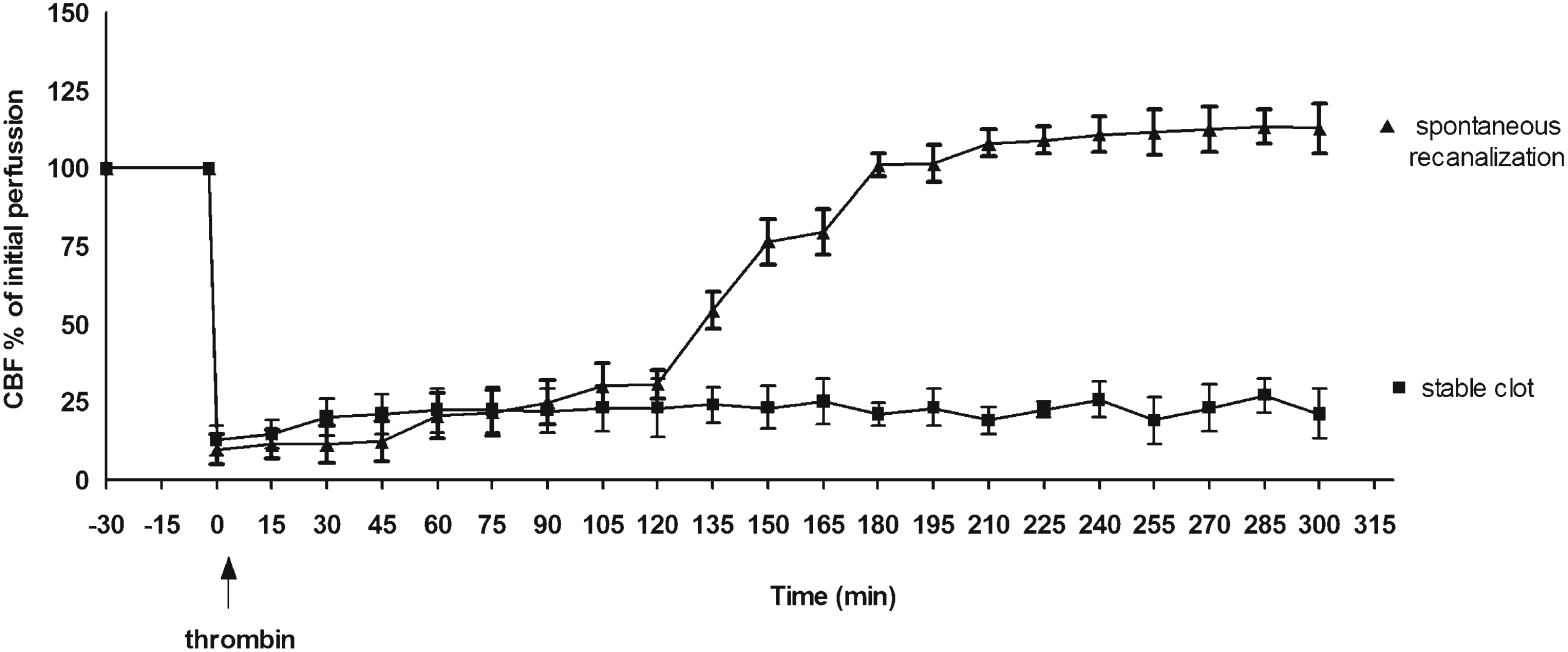
Illustration of clot stability for 1.5 units thrombin. Data were obtained by laser Doppler flowmetry and data are expressed as mean ± s.e.m. values, *n* = 6

A total of 126 surgeries were performed. A total of 82 (65 %) of animals were excluded from the study because of an unsuitable location of the MCA bifurcation for thrombin injection, bleeding complications, or spontaneous reperfusion within 20 min (for details see Fig. 2). Out of the 82 animals, 46 were excluded before thrombin injection. Of the 70 animals that were injected with thrombin, 63 % demonstrated stable clot formation and cortical brain injury (Fig. 3). In 37 % of the animals, we obtained a spontaneous recanalization within 20 min which is equivalent to unstable clot formation (Fig. 2) As a result of injecting the thrombin, the cortical blood flow dropped rapidly to 13 ± 4 % of resting flow. The stability of the clot was studied by laser Doppler measurement up to 5 h after clot formation. The results showed a stable clot up to 2 h after formation, subsequently 20 % of the animals recanalized spontaneously (Fig. 4). In all animals where a stable clot was obtained after 20 min, the clot remained stable up to 2 h. There was no difference in success of clot formation between 1.5 and 3.0 UI of thrombin. All the animals that were further investigated in the study have received thrombin injection. The control group is the contralateral side.

### Infarct Volume

**Fig. 5:**
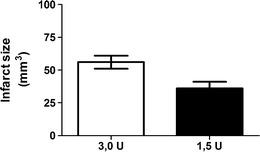
Size of infarct volume. Data are expressed as mean ± s.e.m.; 1.5 units thrombin *n* = 9, 3.0 units thrombin *n* = 9

Injection of thrombin resulted in an infarct that was restricted to the cortex with a mean lesion volume of 36 ± 5 mm^3^ for 1.5 UI and 56 ± 8 mm^3^ for 3.0 UI thrombin (Fig. 5). The infarct size and location was highly reproducible. No edema was detected for any of the groups.

### Histopathological Features

**Fig. 6:**
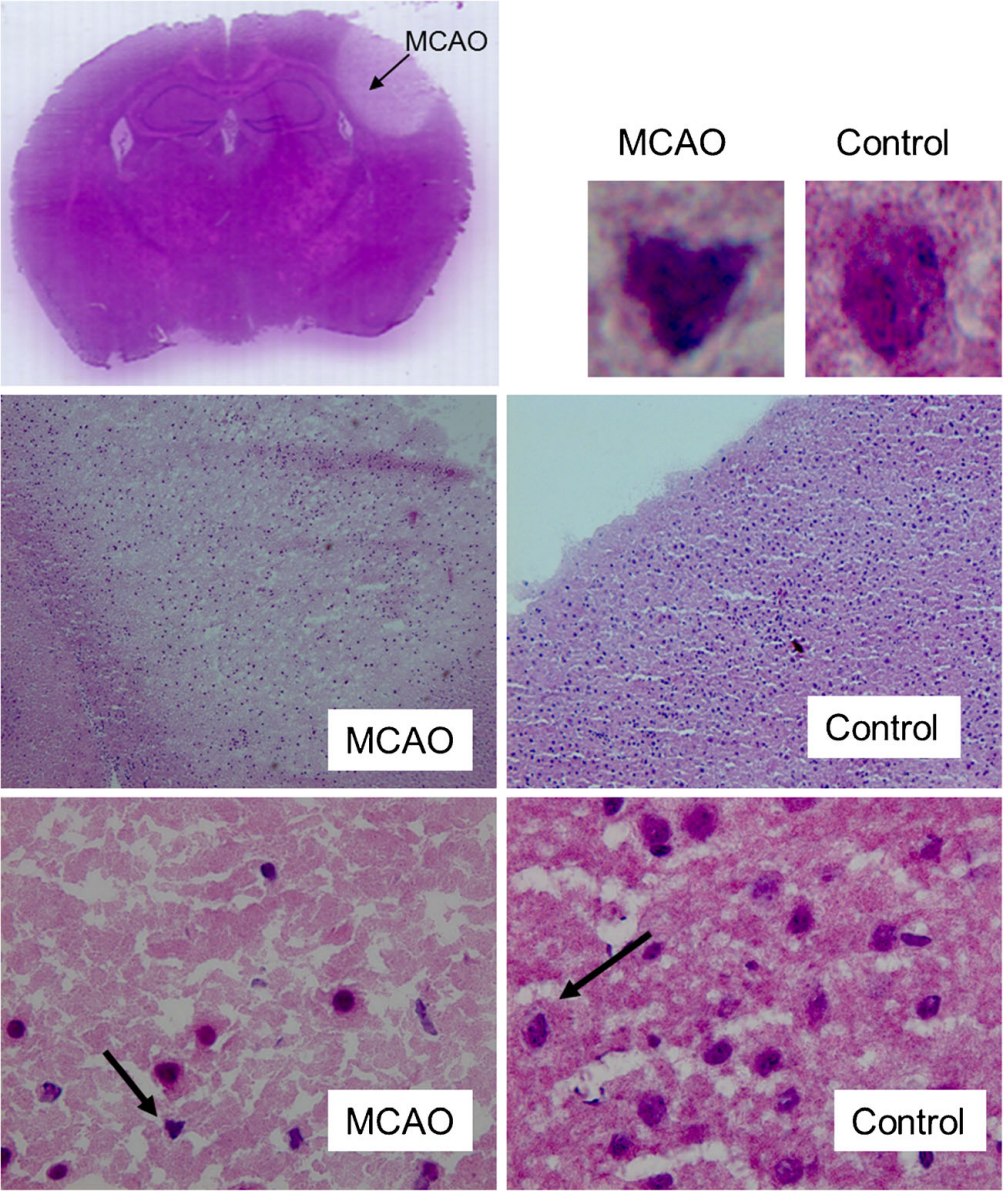
Representative hematoxylin–eosin–saffron (HES) staining for 1.5 units thrombin

The ipsilateral cortex showed massive vacuolation of neutrophils and shrunken, scalloped, and pycnotic neurons (Fig. 6).

### Protein Expression Examined with Immunohistochemistry

**Fig. 7:**
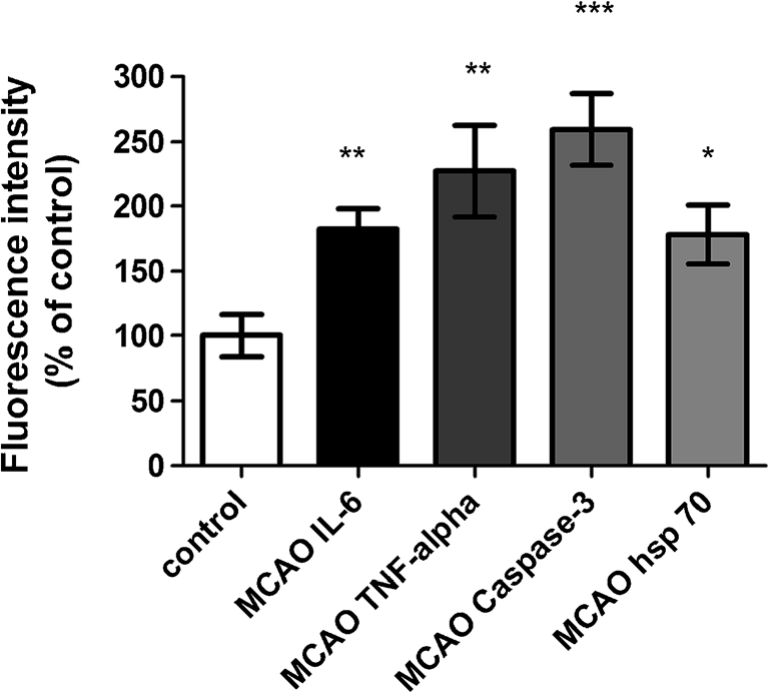
Bar graphs showing semi-quantification of fluorescence intensity for IL-6, TNF-α, caspase 3 and hsp 70 protein levels. Data are presented as the mean percentage relative to control ± s.e.m.; *n* = 9–13, *P ≤ 0.05, **P ≤ 0.01, ***P ≤ 0.001, significant difference between control groups and MCAO. Statistical analyses were performed using Kruskal–Wallis non-parametric test together with Dunn's post-hoc test

**Fig. 8:**
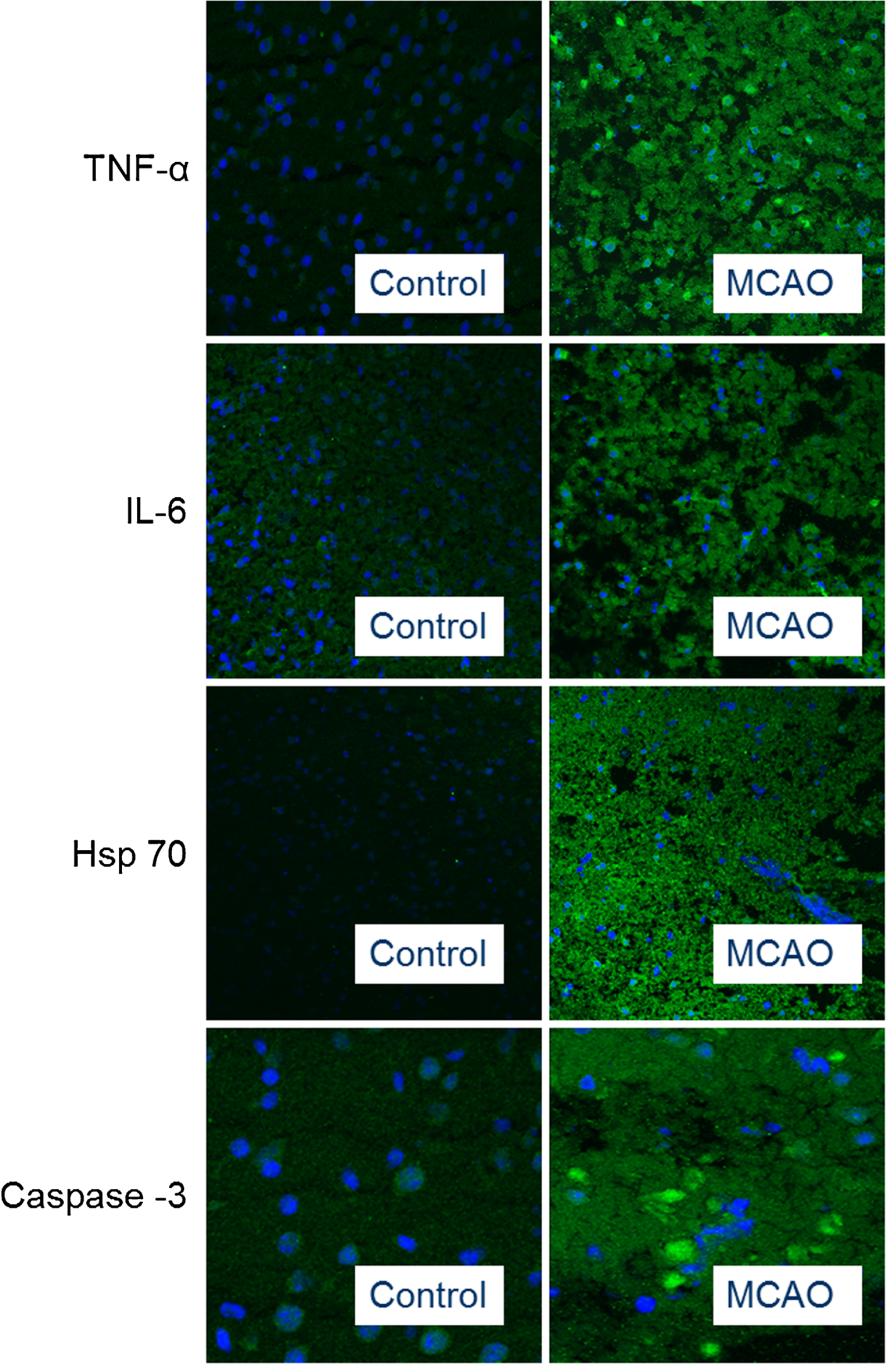
Sections from the brain tissue showing IL-6, TNF-α, caspase 3, and hsp 70 protein expressions. The images represent the control and MCAO. There are significant increases in IL-6, TNF-α, caspase 3 and hsp 70 protein levels in the MCAO group compared to the control groups. Data were obtained with confocal microscopy

The localization and activation of the protein levels were examined by confocal microscopy and immunocytochemistry using selective antibodies towards inflammatory mediators (IL-6, TNF-α), apoptosis (caspase 3) and stress factor (hsp 70). Double immunohistochemistry staining versus nucleus, were performed to verify the localization. The inflammatory mediators IL-6 (182 ± 14 %) and TNF-α (227 ± 30 %) protein levels were increased in the infarct compared to the contralateral side (100 ± 14 %) (Figs. 6 and 7). Similarly, caspase-3 (259 ± 17 %) and hsp 70 (178 ± 21 %) proteins were expressed more in MCAO as compared to control (100 ± 14 %) and (7 %) (Figs. 7 and 8).

## Discussion

In this study, we characterized the model of thromboembolic stroke reported by Orset et al. for C57 black/6J mice [21]. Thrombin injection into the MCA resulted in clot formation in all animals. Cortical infarction occurred in 63 % of the mice while 37 % had a spontaneous MCA recanalization during the first 20 min following thrombin injection. In cases of successful MCA occlusion with consequent infarction, the clot was stable up to 2 h after formation. Subsequently, 20 % recanalized spontaneously. Infarctions were restricted to the cortex with a mean lesion volume of 36 ± 5 mm^3^ for 1.5 UI and 56 ± 8 mm^3^ for 3.0 UI thrombin. Protein levels of IL-6, TNF-α, caspase-3, and hsp 70 were significantly increased after MCAO.

Thus, the model that we have characterized in this study satisfies major criteria for an appropriate animal model for stroke research: (1) it produces a clot formation with accurate and reproducible brain damage, (2) it mimics the human situation where the majority of ischemic strokes results from a sudden occlusion of a blood vessel (most often middle cerebral artery) by a thrombus or embolism, (3) it activates the common cascades such as inflammatory reactions, stress genes, and apoptosis that are normally triggered 24–48 h after stroke [4, 8, 12, 22].

Unfortunately, 65 % of animals were excluded from the study because of an unsuitable location of the MCA bifurcation for thrombin injection, bleeding complications, or spontaneous reperfusion within 20 min. Further obstacles of this model include the necessity for a craniotomy and the difficulty of assessing functional neurological deficits. In this study, we evaluated for the first time this stroke model's effect upon inflammatory processes, stress genes, and apoptosis. In addition, we evaluated the stability of the clot to determine the suitability of the model for investigations of new therapies. The results show that if the clot remains stable during the first 20 min, thrombolytic therapies can be studied up to 2 h without risk for spontaneous recanalization. Interpretation of data later than 2 h after thrombolysis may be difficult, since late spontaneous recanalization may occur (in our study 20 %). Previous investigations have evaluated the new thromboembolic model in Swiss mice[21]. In the present investigation, we have adapted and characterized the model in another strain, the C57 black/6J mouse, which provides new opportunities for studies with transgenic animals. Several studies have demonstrated that the same stroke model may produce variable results in different strains. Thus, it is of great importance that the thromboembolic model developed in Swiss mice is carefully characterized in C57 black/6J mice before performing stroke-related experiments in these animals. The major variation that we could observe with the two strains is that a higher concentration of thrombin is required in C57 black/6J mice to obtain a comparatively similar size of infarction as in the Swiss mice [10, 21] of similar weight. This may be due to differences in the cerebrovascular anatomy of the circle of Willis, which has been shown to result in different susceptibility to cerebral infarction following MCAO in different mouse strains [2]. Elegant studies have been performed showing the high density of collaterals between the ACA and MCA in C57BL/6j mice. However, there is a paucity of such information on Swiss mice. Wang et al. reported that anastomoses between the MCA and ACA in Swiss mice were present at birth but were almost absent within 2 weeks [29]. So we could assume that there are likely significant differences in such anastomoses between C57BL/6J and Swiss mice. It may be that a higher dose of thrombin was required to shut down collateral flow as compared to the Swiss mouse. Furthermore, physical trauma to the MCA during the injection of thrombin could limit blood flow and small clots formed at more distal sites could limit flow in collaterals and the descending arterioles. However, in our histological examinations on animals with stable clots after 20 min, we did not observe a downstream distribution of clots in such specimens. Likewise, we have not seen signs of distal embolism outside the infarct area in 9.4 T MRI monitoring of this procedure (data not shown). Another difference between the models is that C57 black/6J mice seem to be less suitable for the surgical procedure than Swiss mice, due to the location of the MCA bifurcation for thrombin injection, which is often less accessible.

The question of how clot composition can affect treatment efficacy of an occluded vessel is currently a topic of keen interest [18]. Previous analysis of clots produced by the model of thrombin injection in Swiss mice has demonstrated that they consist mainly of polymerized fibrin containing a low number of cells and platelets [21]. This composition is likely different from that of clots in humans. Analysis of clots causing cerebral infarction in humans retrieved by means of the Merci device showed that about 75 % of the clots demonstrated platelet/fibrin accumulation, linear neutrophil/monocyte deposition, and erythrocyte-rich accumulation [25]. Interestingly, the data from this study showed no correlation of thrombus histology with presumed stroke etiology. Moreover, there was no association between thrombus histology and treatment response. One explanation for the difficulty in assessing the response of thrombolytic therapies in terms of clot composition alone may be the highly complex ultrastructure of clots. Electron scanning microscopy has demonstrated a wide variety of thrombi from occluded cerebral arteries in humans with a highly diverse organization of fibrin, platelets, and red cells both within each thrombus and across various thrombi studied [18]. Thus, although our model may not accurately reflect clot composition in human ischemic stroke, it may provide an opportunity for studying the influence of different clot components in the same model, e.g., injection of thrombin vs. injection of FeCL3 (leading to platelet-rich thrombi) on the efficacy of rt-PA thrombolysis. Further studies evaluating thrombolysis with rt-PA in our model will be necessary to validate these contentions.

Brain temperature is a well-known critical variable in stroke research. In the present study, we monitored body temperature with a rectal probe. Our previous data on invasive brain temperature monitoring demonstrated a direct correlation of rectal temperature and brain temperature in rats [9]. When the rat brain temperature rose, there was an equal rise in the rectal temperature. However, this relationship may be more precarious in the mouse. There is some evidence that normal C57 black/6J mice have a slightly lower brain temperature as compared to their rectal temperature and that under intoxication with ethanol, for example, this relationship no longer holds. Whether invasive brain temperature monitoring is necessary in the mouse stroke model may depend upon the type of experiments that are performed. At any rate, should inconsistent results occur when using the model, it would be important to rule out brain temperature as a possible confounding factor.

In summary, the novel thromboembolic model in mice provides a useful tool to stroke researchers in the evaluation of efficacy and safety of thrombolytic therapies and for studies of transgenic animals.
